# Oceanographic Currents and Local Ecological Knowledge Indicate, and Genetics Does Not Refute, a Contemporary Pattern of Larval Dispersal for The Ornate Spiny Lobster, *Panulirus ornatus* in the South-East Asian Archipelago

**DOI:** 10.1371/journal.pone.0124568

**Published:** 2015-05-07

**Authors:** Hoc Tan Dao, Carolyn Smith-Keune, Eric Wolanski, Clive M. Jones, Dean R. Jerry

**Affiliations:** 1 Centre for Sustainable Tropical Fisheries and Aquaculture, James Cook University, Townsville, QLD 4810, Australia; 2 College of Marine and Environmental Science, James Cook University, Townsville, QLD 4810, Australia; 3 TropWATER, James Cook University, Townsville, QLD 4810, Australia; 4 Institute of Oceanography, Vietnam Academy of Science and Technology, 01—Cau Da, Nha Trang, Vietnam; CSIRO, AUSTRALIA

## Abstract

Here we utilize a combination of genetic data, oceanographic data, and local ecological knowledge to assess connectivity patterns of the ornate spiny lobster *Panulirus ornatus* (Fabricius, 1798) in the South-East Asian archipelago from Vietnam to Australia. Partial mitochondrial DNA control region and 10 polymorphic microsatellites did not detect genetic structure of 216 wild *P*. *ornatus* samples from Australia, Indonesia and Vietnam. Analyses show no evidence for genetic differentiation among populations (mtDNA control region sequences *Φ_ST_* = -0.008; microsatellite loci *F_ST_* = 0.003). A lack of evidence for regional or localized mtDNA haplotype clusters, or geographic clusters of microsatellite genotypes, reveals a pattern of high gene flow in *P*. *ornatus *throughout the South-East Asian Archipelago. This lack of genetic structure may be due to the oceanography-driven connectivity of the pelagic lobster larvae between spawning grounds in Papua New Guinea, the Philippines and, possibly, Indonesia. The connectivity cycle necessitates three generations. The lack of genetic structure of *P*. *ornatus *population in the South-East Asian archipelago has important implications for the sustainable management of this lobster in that the species within the region needs to be managed as one genetic stock.

## Introduction

The ornate spiny lobster, *Panulirus ornatus*, lives in tropical waters of the Indo-West Pacific from the Red Sea and south-east Africa in the west to Japan and Fiji in the east.The species is of significant commercial importance supporting local capture fisheries and developing aquaculture operations. *P*.*ornatus* pueruli (final lobster larval stage) are heavily exploited as seed-stock for aquaculture in South-East Asia, particularly in Vietnam and Indonesia, where wild pueruli are collected from the ocean in large numbers [1]. Even though natural fluctuations on larval recruitment are common to other spiny lobsters [2–4], there is concern from fishery managers that large fluctuations in juvenile recruitment and puerulus settlement repeatedly experienced in recent years represent a high risk to the adult lobster fishery [5, 6]. This apprehension was highlighted in 2006–2007 and 2009–2010, when the *P*. *ornatus* pueruli wild harvest in Vietnam was only ~50% of that caught in other years [1, 7]. This high variability in pueruli settlement raised concerns as to whether the annual removal of 1–2 million pueruli by fishers in Vietnam was significantly impacting the demography of the species, particularly that of adult populations. However, very little is understood about the population distribution and dynamics of *P*. *ornatus* and it is not known where the source of pueruli being harvested is situated. An investigation into the connectivity among spiny lobster populations is therefore needed to provide data on the resilience and sustainability of heavy exploitation, as well as to provide information on larvae sources and sinks.

**Fig 1:**
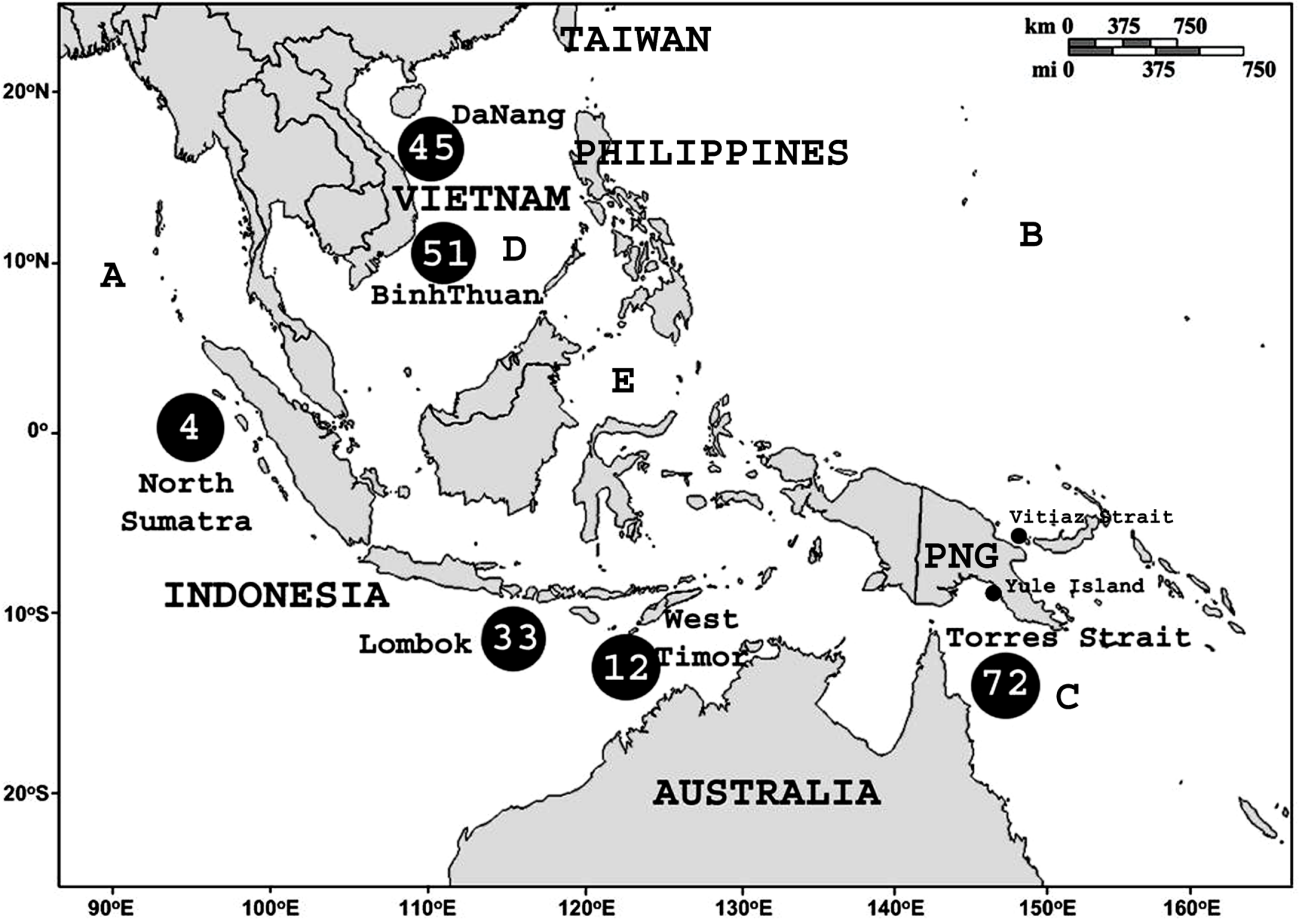
Sampling sites and number of *Panulirus ornatus* specimens collected from across the tropical waters of the South-East Asian archipelago. The numbers of individuals sampled at each location are indicated within the black circles. A Indian Ocean. B Pacific Ocean. C Coral Sea. D South China Sea. E Sulawesi Sea.

Several approaches are available to evaluate connectivity between marine populations, including genetic markers (e.g. mitochondrial DNA or microsatellites), geochemical markers (e.g. microchemical signatures in shells), and/or the utilization of high-resolution biophysical models; however none of these approaches are likely to be conclusive in isolation and have not yet been applied to adequately address population connectivity in *P*. *ornatus* [8–10]. To date the studies that have been done have been limited to hydrodynamic-dispersion models of *P*. *ornatus* in restricted areas of the species distribution. Previous studies in eastern Australian waters [11] and in the Philippines [12] focused on short-term larval dispersion within a single generation of *P*. *ornatus* (i.e. from spawning ground to puerulus settlement site over a few months). According to these models most larvae released from spawning grounds in the Coral Sea, such as the Gulf of Papua, would be carried back to the coastline of northeast Queensland, while a part of them could advect northward to the Vitiaz Strait of eastern PNG within three months [11]. From different spawning grounds in both western and eastern coasts of the Philippines, larvae would be transported northward to Taiwan, advected into the South China Sea, or dispersed into the interior of the Sulawesi Sea [12]. These studies, however, could not address the issue of connectivity of *P*. *ornatus* across the broader South-East Asian archipelago (Fig 1).

The bathymetry and oceanography of the South-East Asian archipelago is very complex, with numerous shoals, straits, islands, reefs, and semi-enclosed seas, as well as mass-flow of water carried by currents between the Pacific and Indian Oceans. Currently there is no fine-scale oceanographic model encompassing the whole archipelago to assist in better understanding the drivers influencing *P*. *ornatus* genetic structure, or in fact connectivity of other marine species. The existing oceanographic models include a number of fine-scale models focusing on restricted areas within the archipelago (e.g. [13]), and medium-scale models of the whole domain (e.g. [14]) that have a grid size too coarse to resolve the current fluxes through the Philippines Straits and, as a result, they largely ignore the connectivity between the Philippine Sea, the South China Sea and the western Pacific Ocean [15].

Adults *P*. *ornatus* are found in waters from 1 to 50m in depth and occupy diverse habitats such as sandy and muddy substrates, coral reefs, rocky bottoms and even turbid coastal waters [16]. Adults are known to migrate by walking along the seafloor for hundreds of kilometers to form large spawning aggregations; for instance, adult *P*. *ornatus* from the Torres Strait, Australia, migrate up to 500 km to a spawning ground near Yule Island in the Gulf of Papua [17–19]. Furthermore, *P*. *ornatus* larvae have a long planktonic phase lasting between 135 to 210 days [17, 20]. Before settlement the larvae metamorphose to the puerulus stage, which is the final larval stage with strong swimming ability; this phase lasts 9–25 days in the laboratory and possibly more in nature [4, 17]. Williams [6]suggested that this long larval development period, swimming ability of puerulus, and the potential for mixing of the phyllosoma in eddies of the South-East Asian archipelago would result in low levels of population genetic structure for the species in the region. However, to date this hypothesis has not been tested.

The present study used molecular genetic techniques to elucidate the genetic population structure of *P*. *ornatus* within the South-East Asian archipelago. To explain the observed patterns of genetic structure, the potential dispersal pathway of *P*. *ornatus* was inferred based on a synthesis of data on regional oceanography and the lobster’s known biology.

## Methods

### Tissue collections and DNA extraction

All work was done in compliance with the Australian Code of Practice for the Care and Use of Animals for Scientific Purposes, and the Queensland Animal Care and Protection Act 2001 under Animal Ethics Permit no. A1746, as approved and administrated by James Cook University Animal Ethics Committee. Animals were collected from commercial aquaculture or fishery operations and no specific collection permits were required. Animals are not listed as endangered or threatened.

A total of 216 *Panulirus ornatus* individuals were sampled from two sites in Vietnam, three in Indonesia, and one in Australia (Fig 1). Specimens from Vietnamese populations were all pueruli, while those from Indonesia were juveniles at different age groups (17 samples at 150 g each collected in October 2009 and 15 samples at 20 g each collected in April 2010). Vietnamese lobster specimens came from the central coastal waters of the Da Nang (16 ^o^N; 108 ^o^E) and Binh Thuan (11 ^o^N; 108 ^o^E) provinces, while Indonesian samples were collected from North Sumatra (1^o^N; 97 ^o^E), Lombok (9 ^o^S; 116 ^o^E) and West Timor (10 ^o^S; 123 ^o^E). Australian samples were collected from wild-caught Torres Strait juveniles (11 ^o^S; 143 ^o^E; 300 g each). All samples (pleopods from adults or abdominal muscle tissue from juvenile lobsters) were preserved immediately in a DMSO-salt preservative solution [21]. Genomic DNA (gDNA) from all lobster samples was extracted from 4 mm^2^ pleopod clips or from the abdominal muscle tissue of juveniles using a modified CTAB protocol [22].

### Mitochondrial DNA (mtDNA) control region

Whole and partial genome sequences including mtDNA control regions of *Panulirus ornatus*, *P*. *gracilis*, *P*. *stimpsoni*, *P*. *japonicus*, and *P*. *inflatus* from the NCBI database were aligned using SEQUENCHER version 4.5 (GeneCode) and primers to amplify 809 base pairs of the mtDNA control region designed based on conserved sites using PRIMER3WEB version 3.0.0 (http://primer3.wi.mit.edu/). These primers were; PO_F2 5’—ATAAAGGTAATAGCAAGAATC and PO_R1 5’—CAAACCTTTTGTCAGGCATC.

Extracted DNA from samples was diluted to 10–40 ng/μl for use in a polymerase chain reaction (PCR). The control region was amplified in 20 μl reaction volumes containing ~5 ng DNA, 1× TM buffer (Qiagen), 1.5 μM of MgCl_2_, 0.2 μM of dNTPs, 0.1 μM of Tag Red (Qiagen) and 0.3 μM of forward and reverse primers. PCR was performed on a BioRadC1000 Thermal Cycler (cycling parameters: 3 min at 95°C, followed by 35 cycles of 95°C for 45 s, 50°C for 30 s, 72°C for 45 s, before a final extension step of 72°C for 5 min). PCR products were then run on a 1.5% agarose gel for quantity and quality verification, and subsequently cleaned-up to remove excess primers by precipitation with isopropanol [23]. A repeat region in the start of the reverse primed sequence resulted in deterioration of sequence. Consequently, only DNA sequence from the forward primer was used. To verify nucleotide base calls each sample was sequenced twice at the Australian Genome Research Facility (AGRF), Brisbane (Australia).

Sequence data were aligned using Geneious ver. 6.0.5 with default alignment parameters and were checked manually for misalignments. Poorly-aligned regions were removed using GBlocks with the default setting [24]. The nucleotide compositions and numbers of variable sites were assessed with MEGA6 [25]. Haplotype and nucleotide diversity for each location were estimated using DNAsp 5.1 [26], while neutrality tests (Tajima's *D* [27]) and partitioning of genetic structure *Φ*_*ST*_ (using genetic distance) as well as pairwise *Φ*_*ST*_ were calculated using ARLEQUIN 3.5 [28]. *Φ*_*ST*_ and pairwise *Φ*_*ST*_ comparisons between populations were estimated using the T92 model (Tamura 1992), with a gamma correction (α = 1.258) as determined by Model Selection in MEGA6 [25]. For calculation of the statistical significance of *Φ*_*ST*_ values obtained, a significance test with 10,000 permutations was undertaken with ARLEQUIN 3.5 [28]. The median-joining network [29] for the haplotypes was constructed using Network v. 4.6.1.0 and Network Publisher v. 2.0.0.1 (http:\\www.fluxus-engineering.com) with default settings.

### Microsatellite markers

Ten highly polymorphic microsatellite markers [30] were used for population genetic investigations. DNA was diluted to 10–40 ng/μl for use as template in a polymerase chain reaction (PCR). Microsatellites were individually amplified in 10 μl reaction volumes containing ~20 ng DNA, 1× Type-it Multiplex PCR Master Mix (Qiagen), 0.04 μM of fluorescent labeled forward primer (TET, FAM or HEX), and 0.2 μM of reverse primer. PCR was performed on a BioRadC1000 Thermal Cycler (cycling parameters: 5 min at 95°C, followed by 28 cycles of 95°C for 30 s, 58°C for 90 s, 72°C for 30 s, before a final extension step of 60°C for 30 min). The PCR products then were checked for consistent amplification by visualization on a 1.5% agarose gel. After this step, PCR products were pooled according to size, fluorescent label, and product quantity and the pooled products were purified using Sephadex G-50 resin, before loading on a Megabace 1000 Capillary Sequencer for size separation of alleles (Amersham Biosciences). Alleles were scored on the basis of fragment size using Fragment Profiler 1.2 (Amersham Biosciences).

Summary statistics such as the number of alleles, as well as observed and expected heterozygosities, were calculated for microsatellites in GENALEX 6.1 [31], which was also used to test for deviations from Hardy-Weinberg Equilibrium (HWE). GENEPOP on the web (http://genepop.curtin.edu.au/) was used to test for linkage disequilibrium among microsatellite loci. Corrections for multiple comparisons (HWE and linkage disequilibrium) were adjusted using the False Discovery Rate (FDR) method [32]. Polymorphic Information Content (PIC) was also calculated for each locus with CERVUS 3.0 [33]. Null allele frequencies were analysed using FreeNA 3.0 [34] while the presence of null alleles and scoring errors were checked using MICROCHECKER 2.2.3 [35].

The level of genetic structure of *P*. *ornatus* based on microsatellite markers was analysed using an Analysis of Molecular Variance (AMOVA) with 10,000 permutations, as well as calculating pairwise *F*_*ST*_ comparisons between populations, both of which were carried out with ARLEQUIN 3.5 [28]. Further to these analyses, the Bayesian clustering algorithm implemented in STRUCTURE ver. 2.3.4 [36] was used to determine spatial genetic discontinuities by inferring the highest probable number of genetic clusters present within the dataset with prior knowledge of the individual’s origin. Individuals are placed in K predetermined sub-groups based on their likelihood of belonging to that sub-group calculated using allele frequencies of multiple loci. K was chosen in advance and ranged from one to 10 and the populations were assumed to be admixed (an individual could belong to any population) in origin. Burn-in and run length were set to 100 000 MCMC (Markov chain Monte Carlo) repetitions and each run was iterated 10 times. This approach implements a model-based clustering method for inferring population structure and assigning individuals to the most probable genetic sub-group or population. Structure Harvester (http://taylor0.biology.ucla.edu/structureHarvester/) was used to determine optimum number of clusters in this analysis. CLUMPP (http://www.stanford.edu/group/rosenberglab/clumpp.html) also was used to average across the replicate run and outputs were entered into DISTRUCT (http://www.stanford.edu/group/rosenberglab/distruct.html) to graph average q values.

The Indonesian samples were a mix of two different age groups (17 samples at 150 g collected in October, 2009 and 15 samples at 20 g collected in April, 2010). To test if there may have been temporally-induced genetic differences among these two collections we first undertook analyses treating each temporal sample as a separate collection. No evidence of genetic differentiation was evident among the temporally separated samples (*Φ*_*ST*_ = -0.0039; *F*_*ST*_ = 0.0037; P >0.05) and accordingly we only report results from analyses for the Indonesian samples where we have treated them as a single population.

### Larval dispersal pathway map

Physical and biological data were integrated to develop a larval dispersal pathway map. A literature review was undertaken and expert opinion from relevant fisheries scientists in Australia, Vietnam and Indonesia was sought to identify data on spawning grounds and pueruli settling locations within the archipelago. One spawning ground is located in the southeast of the Gulf of Papua, Papua New Guinea (PNG), where Torres Strait lobsters spawn during the summer months from November to March [6, 19, 37, 38]. A second cluster of spawning grounds has been identified from the Philippines, where lobsters spawn from May to August [12, 39]. Other information included the observation that (a) 3 month old larvae appear in May-June at the southern tip of PNG [11, 37], and also on the eastern side of the Gulf of Papua also by May-June; (b) the central coast of Vietnam receives arrivals of pueruli in September-December in the North (15^o^ N) and November to January in the South (12^o^ N;[1, 7]); (c) another cohort of pueruli arrives to the Indian Ocean coast of northern Sumatra in November-December (Jones & Priyambodo, unpubl. data); and (d) Lombok receives two cohorts of *P*. *ornatus* pueruli, one cohort arriving in December-February and the second cohort arriving in August-November [1].

This biological data was then merged with oceanographic data to construct a map of the mean surface water circulation in the South-East Asian Archipelago, focusing on different months for different areas based on the known age of lobster larvae found during those months in those areas. We studied only the currents in the surface well-mixed layer, i.e. the layer above the thermocline, which in the tropics is typically about 100 m deep [40]. The *P*. *ornatus* larvae are found mainly within this layer [11] and it is only at the late-stage phyllosoma (i.e. older than 5 months) that the lobster can be found below the thermocline [41].The main data source of currents in the surface well-mixed layer was the ARGO program (http://www.aoml.noaa.gov/phod/argo/introduction_argo.php), but this had limited coverage for the South China, Philippines and Indonesian Seas. For those seas, the results of other field studies (listed in Table 1) were used, together with the results of the previous regional oceanographic models [13, 42–49], and for the South China Sea only (Daryabor, F.; unpubl. data). Streamlines were drawn representing the connectivity between sites where lobster data were available, using Microsoft Visio software v.2003.The length (*L*) between two sites was measured by Distance Calculator using Google Maps (http://www.mapdevelopers.com/distance_finder.php), not as a straight line, but as the length of the streamline of the flow field joining these two sites; and *u* is the average speed of surface ocean current along that streamline during that period. Thus, the estimated time (t) for the larvae to reach different locations was calculated by Fischer [50],

t=Lu (1)

**Table 1:** Field studies providing data of monthly-averaged currents in the surface-well mixed layer for the South-East Asian archipelago.

Author	Data source	Data Period
ARGO (2013)	Ocean drifters	2000–2013
Cravatte *et al*. [43]	ShipbornADCP	1985–2007
Condie [42]	NCEP-NCAR40-year Reanalysis dataset	1982–1997
Forbes and Church [44]	National Aeronautics and Space Administration (NASA)	1978–1979
Liang *et al*.[45]	ShipbornADCP	1997–2001
Manh and Yanagi [46]		
Mayer *et al*. [47]	Data of the World Ocean Atlas	1970–2006
Metzger *et al*.[48]	Digital Bathymetric Data Base 2 (DBDB2)	2004–2006
Potemra and Qu [49]	National Oceanic and Atmospheric Administration (NOAA)	
Schiller *et al*.[13]	ARGO data	1992–2006

ADCP = Acoustic Doppler Current meter.

## Results

### Genetic variation of the mtDNA control region

Nucleotide sequences of the control region were determined for 189 *P*. *ornatus* individuals (Genbank accession no. KJ956062-KJ956250). A small number of samples for which DNA was extracted failed initial quality control checks and were not successfully sequenced. From the 189 individuals sequenced successfully a total of 182 haplotypes were detected, with 601 sites without gaps and missing data and 231 were polymorphic (Table 2). Among 7 shared haplotypes, only one was shared among individuals at the same sampling site, six other haplotypes were represented at two sampling sites (S1 Table).

**Table 2:** Genetic indices for mtDNA control region characterized in *Panulirus ornatus* from six sample sites/collections.

Pop	*N*	*H*	*hd*	*Pi*	Tajima's *D (p-value)*
**Torres Strait**	54	51	1.000 ± 0.004	0.028 ± 0.002	-1,473 (0.088)
**West Timor**	11	11	1.000 ± 0.039	0.029 ± 0.006	-1.200 (0.136)
**Lombok**	28	27	0.997 ± 0.010	0.034 ± 0.004	-1.521 (0.088)
**North Sumatra**	4	4	1.000 ± 0.177	0.020 ± 0.005	-0.727 (0.279)
**Binh Thuan**	51	51	1.000 ± 0.004	0.028 ± 0.003	-1.859 (0.050)
**Da Nang**	41	41	1.000 ± 0.005	0.026 ± 0.003	-1.497 (0.088)
**Total/Mean**	189	182	1.000 ± 0.001	0.028 ± 0.001	-1.379 (0.089)

*N*, sample size; *H*, number of haplotypes; *hd*, haplotype diversity; *Pi*, nucleotide diversity;

The *P*. *ornatus* mtDNA control region was found to exhibit an extremely high mutation rate, resulting in high haplotypic diversity whereby almost every lobster individual possessed a unique haplotype (Table 2). Haplotype diversities ranged from 0.997 to 1.000 within populations, while nucleotide diversities indicating the degree of polymorphism within a population/ sample collection ranged from 0.020 (North Sumatra) to 0.034 (Lombok).

**Fig 2:**
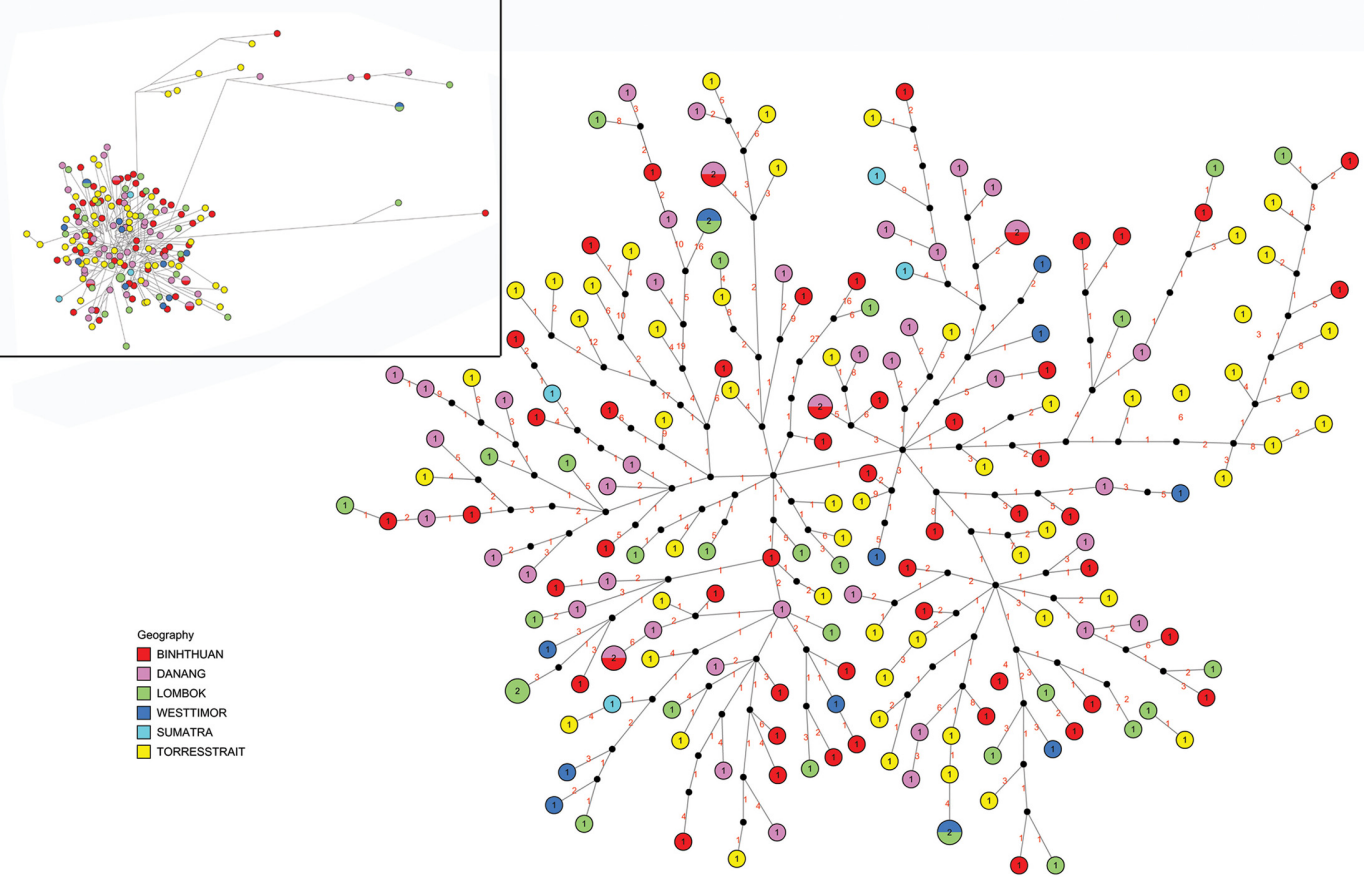
Haplotype network of *Panulirus ornatus* control region sequences from six collection locations from the South-East Asian archipelago. The larger tree on the right has been edited to show more detail, and the unedited tree is shown in the inset. Each circle represents a haplotype, whose diameter is proportional to the number of individuals with that haplotype. The black dots on the lines between haplotypes represent missing haplotypes. The numbers on the connecting lines are the number of mutations between haplotypes.

No significant population subdivision was detected among the populations sampled, with a non-significant fixation index evident (*Φ*_*ST*_ = -0.002; P = 0.648). All of the genetic variation measured with mtDNA occurred within populations, with no detectable among-population variance (Table 3). In addition, no evidence of individual population-level genetic structure was detected across the wide geographical range sampled from the Torres Strait of Australia to Vietnam and Indonesia, with negligible and non-significant pairwise *Φ*_*ST*_ values between populations being very low (from -0.042 to 0.010, P >0.05) (Table 4). Similar evidence for a lack of structuring was supported by the Tajima's *D* values, which were not significant at all localities (from -1.521 to -0.727, P >0.05) (Table 2). As further evidence for widespread gene flow and lack of genetic structure the haplotype network tree showed no clustering of haplotypes into geographical regions, or location based groups, with the majority of haplotypes being single or unique units (Fig 2). Therefore, mtDNA analyses based on the control region provided no evidence for genetic population structure in *P*. *ornatus* across the geographical range sampled.

**Table 3:** Summary table of analysis of molecular variance (AMOVA) describing the partitioning of genetic variation for six *Panulirus ornatus* populations based on both mtDNA control region sequences and 10 microsatellite loci.

	Source of variation (%)			
	Among population	Within population	*Φ*_*ST*_/*F*_*ST*_	*p- value*
**mtDNA-control region**	-0.20	100.20	-0.002	0.648
**Microsatellites**	0.26	99.74	0.003	0.195

**Table 4:** Genetic differentiation between *Panulirus ornatus* from collection locations using pairwise *Φ*_*ST*_ for mtDNA-control region (upper value) and pairwise *F*_*ST*_ for microsatellite loci (lower value).

	Australia	Indonesia			Vietnam	
Localities	Torres Strait	West Timor	Lom-bok	North Sumatra	Binh Thuan	Da Nang
Torres Strait		0.007	0.005	-0.012	0.002	0.010
West Timor	0.006		-0.030	-0.038	-0.005	-0.012
Lombok	-0.003	0.008		-0.042	-0.008	-0.004
North Sumatra	0.029	0.030	0.034		-0.037	-0.026
Binh Thuan	0.001	0.002	0.004	0.026		-0.006
Da Nang	0.000	0.009	0.001	0.017	0.002	

No significant value was found after correction using FDR.

### Genetic variation of microsatellite markers

Ten polymorphic microsatellite markers were successfully amplified (Table 5) and PCR products of all 216 samples of *P*. *ornatus* were genotyped for subsequent population genetics analyses. A total of 143 alleles were observed, ranging from five (Orn_01) to 29 (Orn_11) alleles per locus. Significant departures from HWE were observed for two loci in the Lombok and Binh Thuan populations (Orn_01 in Lombok and Orn_17 in Binh Thuan) and the dataset was reanalyzed with and without these markers in these two populations to test if they were significantly influencing results obtained. No differences were found in genetic structure indices when these markers were included so the complete dataset of markers were analyzed and is presented here. No linkage disequilibrium was detected among the 10 loci genotyped. Null allele frequencies were above 10% at locus Orn_02 in Lombok (13%), at locus Orn_17 in West Timor (17%) and in Binh Thuan (15%) (S2 Table). Null allele frequencies also detected at loci Orn_16 (17%) and Orn_21 (11%) in North Sumatra, which could be the results of small samples size (4 samples). Dataset then were corrected and reanalysed. The results showed no difference in the AMOVA test and pairwise *F*_*ST*_ estimates (S3 and S4 Table). The original dataset was therefore left unchanged.

**Table 5:** Genetic indices for 10 microsatellites characterized in *Panulirus ornatus* at six sample sites/collections.

		Microsatellites									
Localities		Orn 01	Orn 02	Orn 11	Orn 12	Orn 16	Orn 17	Orn 18	Orn 20	Orn 21	Orn 25
**Torres Strait**	***N***	72	72	71	71	71	70	72	69	71	70
	***N***_***A***_	8	4	20	23	9	10	9	10	7	9
	***H***_***O***_	0.72	0.54	0.85	0.94	0.79	0.5	0.74	0.42	0.79	0.71
	***P***_***HWE***_	0.98	0.93	0.98	0.76	0.98	0.98	0.98	0.98	0.92	0.98
**West Timor**	***N***	12	12	12	11	12	12	12	12	12	12
	***N***_***A***_	6	3	13	9	6	7	9	7	6	7
	***H***_***O***_	0.75	0.58	0.92	0.91	0.75	0.42	1	0.67	0.83	0.83
	***P***_***HWE***_	0.95	0.43	0.93	0.98	0.98	0.32	0.93	0.98	0.95	0.98
**Lombok**	***N***	32	32	32	32	32	31	32	30	32	32
	***N***_***A***_	10	3	20	19	8	7	10	7	8	7
	***H***_***O***_	0.69	0.41	0.88	1	0.84	0.45	0.69	0.3	0.72	0.63
	***P***_***HWE***_	**0.00**	0.32	0.11	0.98	0.56	0.95	0.98	0.98	0.98	0.93
**North Sumatra**	***N***	3	3	3	3	4	3	3	3	3	3
	***N***_***A***_	3	3	5	4	5	2	5	1	3	3
	***H***_***O***_	0.33	0.67	1	1	0.5	0.67	1	0	0	1
	***P***_***HWE***_	0.93	0.95	0.95	0.98	0.75	0.93	0.98		0.97	0.93
**Binh Thuan**	***N***	49	49	49	48	49	51	10	49	51	50
	***N***_***A***_	8	4	22	21	7	6	6	9	7	9
	***H***_***O***_	0.69	0.67	0.9	0.94	0.76	0.33	0.7	0.55	0.73	0.74
	***P***_***HWE***_	0.93	0.56	0.68	0.98	0.95	**0.00**	0.58	0.98	0.58	0.98
**Da Nang**	***N***	40	40	44	44	44	43	0	45	44	45
	***N***_***A***_	8	3	24	21	8	10	0	8	8	10
	***H***_***O***_	0.75	0.65	0.91	0.91	0.82	0.67	0	0.47	0.75	0.73
	***P***_***HWE***_	0.78	0.95	0.98	0.43	0.98	0.98		0.98	0.95	0.98
**Mean**	***N***	34.67	34.67	35.17	34.83	35.33	35.00	21.50	34.67	35.50	35.33
	***N***_***A***_	7.3	3.5	17.2	16.0	7.3	6.8	6.3	7.0	6.33	7.3
	***H***_***O***_	0.70	0.60	0.91	0.89	0.74	0.49	0.69	0.39	0.69	0.77
	***PIC***	0.69	0.55	0.92	0.93	0.72	0.54	0.71	0.44	0.81	0.69
**Total**	***N***	208	208	211	209	212	210	129	208	213	212
	***N***_***A***_	11	5	29	26	11	16	11	14	8	12
	***Allele size range (bp)***	139–176	258–278	175–242	304–400	163–195	260–321	352–372	318–362	240–264	184–216

*N* sample size, *N*_*A*_ number of alleles, *H*_*O*_ observed heterozygosity, *P*_*HWE*_ Hardy-Weinberg equilibrium significance value at P<0.05 after FDR correction, *ns* non-significant, *bold text* significant, *PIC* polymorphic information content.

As for the mtDNA control region, no significant population genetic structure was evident between the six sites when genotyped with the 10 microsatellite loci. *F*_ST_ estimates of population structure were again negligible and non-significant (AMOVA, *F*_ST_ = 0.003; P = 0.195) (Table 3). The microsatellite data indicated that less than 1% of genetic variation was present among populations. A similar lack of population genetic structure was evident in pairwise population comparisons across the Indo-West Pacific region (*F*_ST_ ranged from -0.003 to 0.031, Table 4). The pairwise *F*_ST_ comparison between North Sumatra and other sampling sites were the highest observed (from 0.017 to 0.034), but were all non-significant after FDR correction (P > 0.05). Due to the small sample size collected from North Sumatra the higher sample *F*_ST_ values involving this population are likely a result of random sampling effects and small sample size.

**Fig 3:**
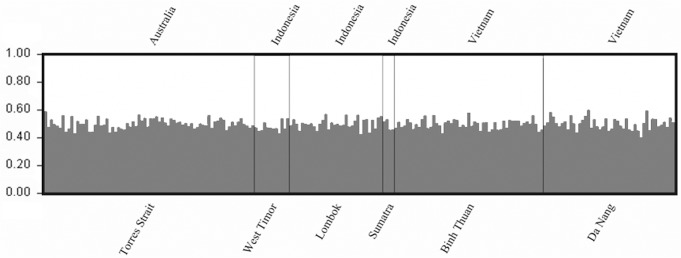
Bayesian individual assignment analysis for K = 2 for *Panulirus ornatus* genotyped at ten microsatellites across six Indo-Pacific sampling sites. Colours (grey or white) represent probability (y-axis) of individuals being assigned to each genetic cluster, whilst numbers (x-axis) represents population individuals sampled from 1 = Torres Strait, 2 = West Timor, 3 = Lombok, 4 = North Sumatra, 5 = Binh Thuan (Vietnam), 6 = Da Nang (Vietnam). Sampling locations were used as priors.

Individual based Bayesian assignment tests supported the lack of population genetic structure indicated by non-significant and small pairwise *F*_ST_ estimates. Although Structure Harvester suggests K = 2 from multiple simulations runs at values of K from 1 to 10, visual examination of individual bar plots for K = 2 indicates an inability of the STRUCTURE algorithm to reliably assign any of the individuals to a distinct cluster, with assignment probabilities for each of the two populations of ~50% for all individuals sampled in each of the 10 replicate runs (Fig 3). The inability to assign individuals using post-hoc plots if the true K < 2 has been discussed in Evanno *et al*. [51]. Therefore, Bayesian analysis using STRUCTURE, also suggests a lack of genetic structure, among the six populations examined, despite the widely spaced regional sampling employed here.

### Larval dispersal pathway map throughout the South-East Asian archipelago

**Fig 4:**
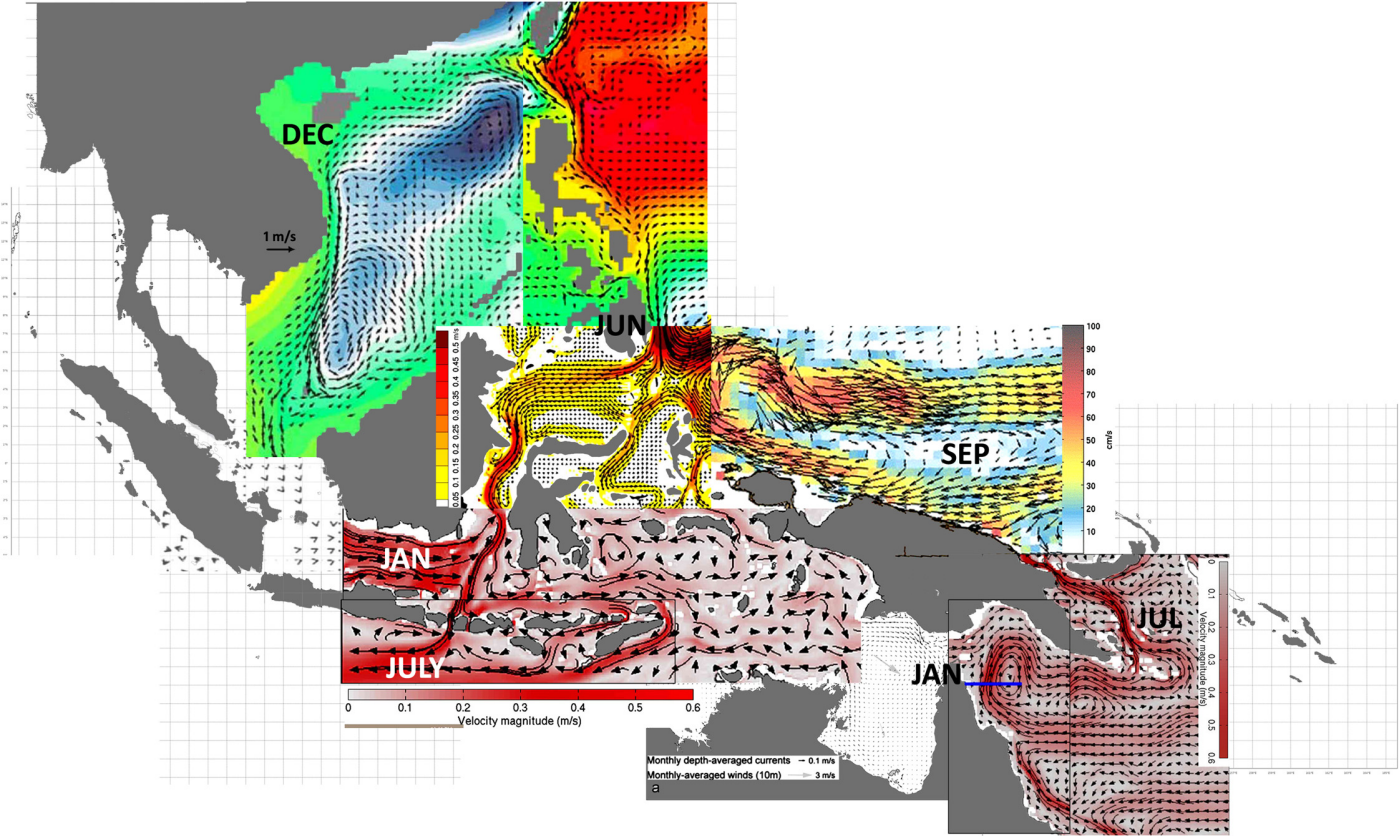
Seasonal surface ocean currents in the surface well-mixed layer in the South-East Asian archipelago at the times when *Panulirus ornatus* larvae are travelling between the various sites shown in Fig 5, based on previous studies [13, 42, 45, 48, 49] and ARGOS data (http://www.aoml.noaa.gov/phod/graphics/dacdata/seasonal_wpac.gif). To explain how this connectivity may eventuate, this figure shows the distribution of currents in the surface well-mixed layer in the South-East Asian archipelago during the periods of larval transport listed in Table 6.

**Fig 5:**
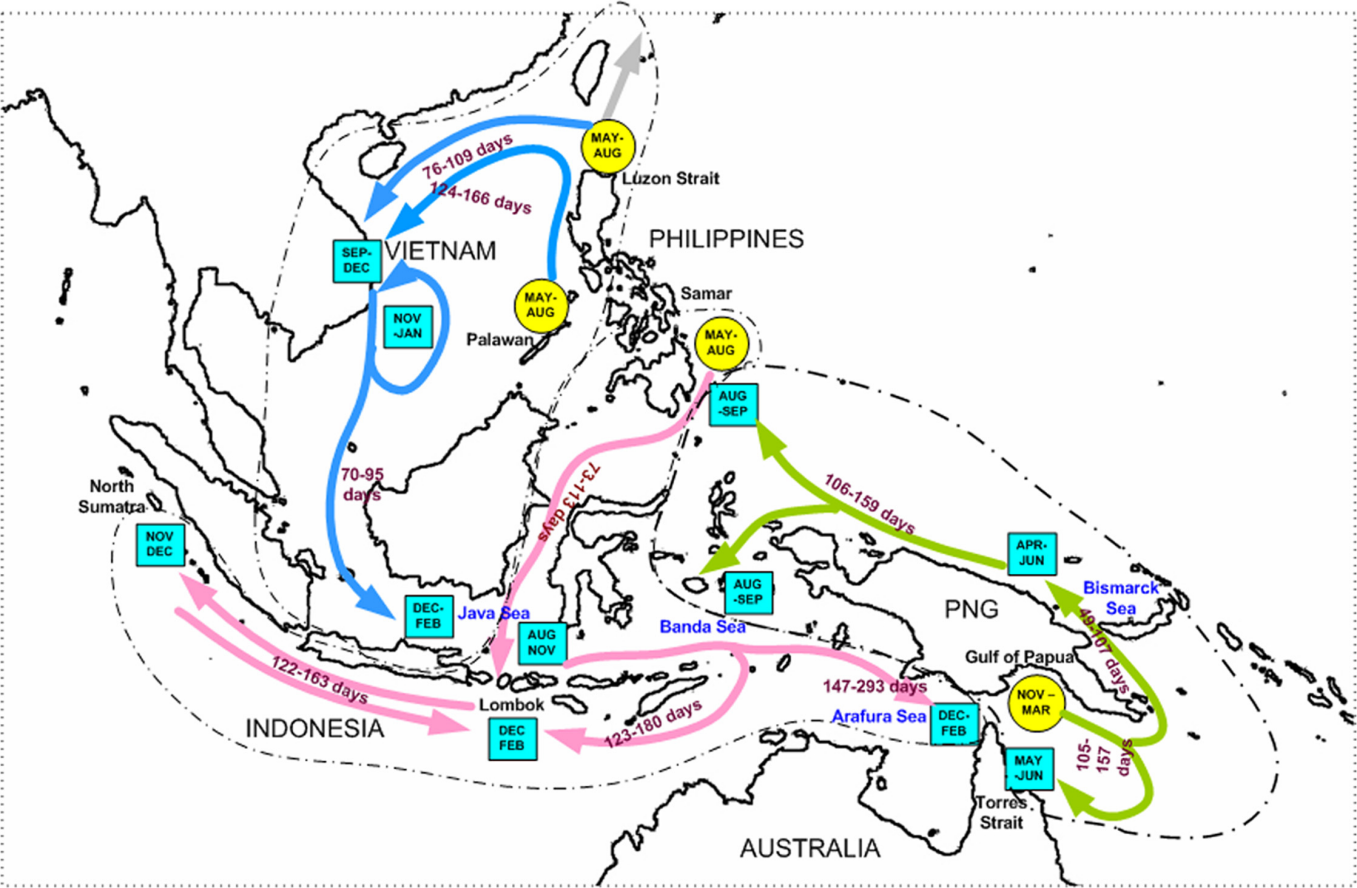
Suggested larval dispersal pathways based on the surface water oceanography and the location of spawning grounds, dispersion patterns and connectivity for *Panulirus ornatus* larvae throughout the South-East Asian archipelago. Round circles indicate spawning grounds where the larvae are released. Square boxes are estimated time that the larvae reach different locations as suggested by the oceanography and confirmed by field data; out of them only two points have no field data, namely arrival times of larvae from Lombok in Torres Strait and the time of transit of the larvae along the north coast of Papua New Guinea. The different colours represent the different putative larval dispersal pathways, in order to distinguish separate flows. Estimated time is presented in Table 6.

The above genetic studies using both mtDNA control region and microsatellites reveal a single genetic population of *P*. *ornatus* within the South-East Asian archipelago, implying high population connectivity of *P*. *ornatus* throughout this region. To explain how this connectivity may eventuate, the distribution of currents in the surface well-mixed layer in the South-East Asian archipelago is shown in Fig 4 for the larval transport periods listed in Table 6. From these data, the suggested connectivity network is shown in Fig 5 and further elaborated on in the discussion. Accordingly, the apparent lack of genetic structure in this tropical lobster species across South-East Asian archipelago is explained by current-mediated larval transport that connects lobsters among spawning populations. This connectivity requires at least three generations.

**Table 6:** Estimate time (*t*) for larvae/pueruli to reach different locations in the South-East Asian Archipelago, calculated from the length (*L*) between two sites, measured by Distance Calculator (http://www.mapdevelopers.com/distance_finder.php) and the average speed of current (*u*) in the surface well-mixed layer along that streamline during that period, inferred from modelling studies of different authors.

Route	Local Route	Author	Data time	Data depth	*u* (cm/s)		*L*	*t* (days)		
				(m)	From	To	(km)	From	To	Average
PNG -	Gulf of Papua—Torres Strait	Schiller *et al*. [13]	Jan-Jul	< 250	10	15	1,361	105	157	105–157
Australia -	Gulf of Papua—Bismack Sea	Schiller *et al*. [13]	Jan-Jul	< 250	15	30	1,381	53	107	49–107
Philippines		Cravatte*et al*. [43]	Jan-Jul	< 100	15	35	1,381	46	107	
	Bismack Sea—Philippines/Banda Sea	ARGO (2013)	Jun-Sep	< 50	20	30	2,744	106	159	106–159
	Luzon Strait—Central Vietnam	Manh and Yanagi [46]	Oct-Dec	~ 0	15	20	1,416	82	109	76–109
Philippines -		Liang *et al*. [45]	Dec	< 50	15	20	1,416	82	109	
Vietnam -		Potemra and Qu [49]	Dec-Jan-Feb	< 100	15	25	1,416	66	109	
Indonesia	Palawan—Central Vietnam	Liang *et al*. [45]	Dec	< 50	15	20	2,148	124	166	124–166
	Central Vietnam—Java Sea	Liang *et al*. [45]	Dec	< 50	25	30	1,818	70	84	70–95
		Farshid (unpubl. data)	Dec-Jan-Feb	< 10	20	30	1,818	70	105	
	Eastern Samar—Lombok	Liang *et al*. [45]	Jul	< 50	25	50	2,674	62	124	67–119
		Schiller *et al*. [13]	Jul	< 250	25	45	2,674	69	124	
		Potemra and Qu [49]	Mar-Apr-May	< 100	30	40	2,674	77	103	
		Metzger *et al*. [48]	Mean	< 120	25	50	2,674	62	124	
Philippines -	North Lombok—South Lombok	Schiller *et al*. [13]	Jul	< 250	20	25	2,663	123	154	123–180
Indonesia-	through Banda Sea	Mayer *et al*. [47]	Oct	< 700	15	25	2,663	123	205	
Australia	South Lombok—North Sumatra	Schiller *et al*. [13]	Jul	< 250	15	20	2,114	122	163	122–163
		Potemra and Qu [49]	Jun-Nov	< 100m	15	20	2,114	122	163	
	Java Sea—Arafura Sea	Forbes and Church [44]	Jan	< 20	10	20	2,536	147	293	147–293
		Schiller *et al*. [13]	Jan	< 250	10	20	2,536	147	293	
		Condie [42]	Jan	< 18	10	20	2,536	147	293	

## Discussion

### Genetic population structure

A combination of mitochondrial DNA control region and microsatellite DNA data suggest a single, genetically homogeneous stock of tropical ornate spiny lobster across a broad region of the South-East Asian archipelago. Genetic differences were not detected between samples of *P*. *ornatus* from Vietnam, Indonesia and Australia-PNG, supporting the hypothesis by Williams [6] of low genetic structuring of this species across the region due to its long oceanic larval development phase and wide larval transport capability. Neither population genetic (F_*ST*_, *Φ*_*ST*_, Bayesian) or phylogeographic network analyses indicated any evidence for restrictions on gene flow across the region and integration of biological and oceanographic data show that genes can potentially circulate unimpeded throughout the entire region in only a few generations.

Similar occurrences of high population inter-connectivity have been observed in other *Panulirus* species and may be a consequence of the long larval dispersal and adult migration life-history within this genus of lobsters. For instance, genetic studies on Japanese spiny lobster, *P*. *japonicus*, failed to reveal any stock heterogeneity within the Japan Sea [52]. Low heterogeneity was also observed for *P*. *gilchristi* in South Africa [53] and *P*. *cygnus* in Western Australia [54]. However, the long pelagic larval duration of *Panulirus* lobsters does not always lead to low population divergence. In *P*. *argus*, for example, genetic differentiation is present between Bermuda and Florida populations within the Caribbean Sea, as well as those from Venezuela and Brazil [55]. Likewise, South African *P*. *delagoae* and *P*. *elephas* populations in the Atlantic Ocean and Mediterranean Sea exhibit shallow, but significant, levels of genetic structuring [56, 57]. Recently, high gene flow was found in *P*. *penicillatus* within localities in Western Pacific, but genetic structure was detected between Western and Eastern Pacific populations [58, 59]. In both study cases, the patterns of ocean currents were considered to be main factors contributing to larval dispersal and thus the population structure of spiny lobster species. Therefore, while life-history might play a part in determining genetic structure in this genus of lobsters, local oceanographic and other biogeographic factors largely drive the level of genetic structure that can be formed.

Regarding the mtDNA data presented in our study, previous papers have illustrated the point that inadequate sampling can lead to the wrong conclusions and for studies based on the mtDNA control region alone, in the situation where such high haplotypic diversity is evident within a species very large sample sizes can be required to detect common haplotypes and associate them with particular geographic areas [60, 61]. As a result, any conclusions made on levels of genetic structure based purely on the mtDNA control region data presented herein should be made with caution.

### Connectivity network and dispersal pathway

Our oceanographic informed dispersal modelling suggests the potential for complete connectivity of *P*. *ornatus* populations within the South-East Asian archipelago within three generations of breeding. Starting arbitrarily as the first generation from the spawning ground in the Gulf of Papua, with spawning known to occur from November to March (Fig 5), modelling suggests currents in this region would carry and split the resultant *P*. *ornatus* larva into two larval plumes; one plume is transported in a loop in the northwest Coral Sea to return to the Torres Strait, while the other plume exits the Coral Sea through the Vitiaz Strait to enter the Bismarck Sea where larvae are carried north-westward along the north-eastern coast of PNG from April to June. Timing is critical for the larvae of this second plume. If the larvae were to arrive two months earlier along the north-eastern coast of PNG, they would be swept eastward into the Pacific Ocean by the Northern Equatorial Counter Current (NEC). Instead, larvae are carried through to the southern Philippines where they are dispersed widely by complex, swift currents through straits between islands from August to September (days 106 to 159 after spawning). This dispersal is facilitated by the directional swimming towards the shore of the pueruli during the last 25 days of development. After metamorphosis to the puerulus stage, lobsters settle to a benthic existence, where they progressively grow to maturity. It is unlikely they will move any great distance during this period, however, upon maturation adult lobsters may migrate up to a few hundred kilometres to the identified spawning grounds in the northern or western Philippines where they will spawn the second generation of larvae [4], [19, 61].

The second generation of larvae are produced from these adults on the east and west coasts of the Philippines from May to August. These larvae then potentially disperse in three plumes. The first plume is advected northward towards Taiwan (Fig 5). The second plume is transported into the South China Sea and reaches the central coast of Vietnam by September to December. This predicted timing of the arrival of pueruli in Vietnam given the time of spawning in the Philippines and ocean current models agrees well with field observations [1, 7]. Part of this larval plume is further transported past Vietnam southward to Indonesia to reach Lombok 70–95 days later. The third larval plume originates from spawning grounds near Samar from May to August, from where newly-spawned *P*. *ornatus* larvae then would rapidly travel with the Mindanao Current southward to Lombok which they would reach in about 73–113 days. At that time the larvae are old enough to metamorphose into pueurli and settle and mature to become the third generation of adults. Spawning *P*. *ornatus* adults in Indonesia appear to produce two larval plumes. One plume originates in the south of Lombok and would travel with the Indonesian Throughflow to arrive in the Indian Ocean and ultimately to reach the Indian Ocean coast of northern Sumatra in November—December. These larvae are advected back towards Lombok during the northern winter monsoon (from November to February) before settling. The second larvae plume originates from the north of Lombok and is advected by currents to the Banda Sea and the northern Arafura Sea during the northern winter monsoon to reach Torres Strait waters by February. Evidence for these small juveniles (<40 mm carapace length) have been observed in Western Torres Strait [62]. We suggest that they will remain there until they mature as adults and can walk eastward across the Torres Strait to spawning grounds in the Gulf of Papua. The connectivity cycle is thus completed over three generations. Thus, based on the timing of the arrival of pueruli cohorts in Lombok and the time expected for dispersal of lobster larvae from Lombok to Torres Strait, we suggest that a third spawning ground exists in Indonesia, possibly around Lombok, but no field data are available. Likewise not all dispersal pathways are understood. Indeed some larvae originating from the Gulf of Papua may be transported southward towards the Java and Banda Seas, although there are no field data to confirm this suggestion.

In our study area the mean sea level was 100 m lower at the end of the last Ice Age about 20,000 years ago. At that time the Torres Strait was land and there was no connection between the Arafura Sea and the Coral Sea; in fact it is only about 8000 years ago that Torres Strait was flooded [63]. Thus the alternative explanation for our observations, namely that there was a formerly widespread population that subsequently became genetically differentiated, but with no apparent genetic signal yet due to incomplete sorting, appears unlikely.

### Implications for management

The existence of a single genetic population of *P*. *ornatus* characterised by drift connectivity [64] might have important implications for the sustainable management of this lobster in that the species within the region needs to be managed as one genetic stock. However, more work is required on the demographic connectivity of these populations so that the combined genetic and demographic connectivity datasets can inform management of this species as either one unit, or on the basis of individual spawning grounds [65].Consequently, a multi-governmental fishery policy should be developed by Australia, Papua New Guinea, the Philippines, Vietnam and Indonesia, to ensure sustainability. While the sinks of *P*. *ornatus* larvae are known, the knowledge of larval sources is still rudimentary, with to date only a few spawning sites confirmed. The present study suggests that an additional spawning ground may also be present in Indonesia, and its location needs to be identified and protected. More detailed studies on population connectivity are necessary to ensure the sustainability of lobsters in the South-East Asia archipelago. Genetic connectivity should be conserved as a priority.

## Conclusion

This study based on population genetic analyses at both mtDNA and nuclear DNA markers indicates high levels of connectivity among *P*. *ornatus* populations throughout the South-East Asian archipelago. The study is novel in terms of population dynamics because it suggests that this connectivity requires three generations for the cycle to complete and is reliant on timing of spawning events, time to settlement and prevailing ocean currents. These results have implications for fisheries management in the region, because there appears to be single stock of *P*. *ornatus*, which requires the engagement of governments and agencies to provide effective management policies for the benefit of all countries. A modelling study of larval transport processes over at least three generations is necessary to better locate the larval dispersal pathways and quantify the population dynamics including the relative influence of self-seeding versus broadcast connectivity between spawning populations.

## Supporting Information

S1 Table Spatial distribution of control region haplotypes among *Panulirus ornatus* from six localities in the South-East Asian archipelago. http://dx.doi.org/10.5061/dryad.sp418/2.
(DOCX)

S2 Table Null allele frequencies in the original dataset and after correction calculated by using FreeNA 3.0. Values above 10% are in bold. http://dx.doi.org/10.5061/dryad.sp418/3.
(DOCX)

S3 Table Summary table of analysis of molecular variance (AMOVA) describing the partitioning of genetic variation for six *Panulirus ornatus* populations in the original dataset and after correction based on 10 microsatellite loci. http://dx.doi.org/10.5061/dryad.sp418/4.
(DOCX)

S4 Table Genetic differentiation between *Panulirus ornatus* from collection locations using pairwise *F*_*ST*_ for microsatellite loci in original dataset (lower value) and after correction (upper value). No significant value was found after correction using FDR. http://dx.doi.org/10.5061/dryad.sp418/5.
(DOCX)
