# Supplementary material for: Oceanographic Currents and Local Ecological Knowledge Indicate, and Genetics Does Not Refute, a Contemporary Pattern of Larval Dispersal for The Ornate Spiny Lobster, Panulirus ornatus in the South-East Asian Archipelago
Source: PLoS One. 2015 May 7;10(5):e0124568. doi: 10.1371/journal.pone.0124568 (PMC4423998; doi:10.1371/journal.pone.0124568)
Supplement: S1 Table — http://dx.doi.org/10.5061/dryad.sp418/2. (DOCX) [file pone.0124568.s001.docx]

Table S1. Spatial distribution of control region haplotypes among *Panulirus ornatus* from six localities in the South-East Asian archipelago
