# Supplementary material for: Oceanographic Currents and Local Ecological Knowledge Indicate, and Genetics Does Not Refute, a Contemporary Pattern of Larval Dispersal for The Ornate Spiny Lobster, Panulirus ornatus in the South-East Asian Archipelago
Source: PLoS One. 2015 May 7;10(5):e0124568. doi: 10.1371/journal.pone.0124568 (PMC4423998; doi:10.1371/journal.pone.0124568)
Supplement: S2 Table — Values above 10% are in bold. http://dx.doi.org/10.5061/dryad.sp418/3. (DOCX) [file pone.0124568.s002.docx]

Table S2. Null allele frequencies in original dataset and after correction calculated by using FreeNA 3.0. Values above 10% are in bold.

|  | **Markers** | **Localities** | | | | | |
| --- | --- | --- | --- | --- | --- | --- | --- |
|  |  | **Torres Strait** | **West Timor** | **Lombok** | **North Sumatra** | **Binh Thuan** | **Da Nang** |
| **In original dataset** | Orn_01 | 0.00 | 0.00 | 0.00 | 0.00 | 0.01 | 0.01 |
|  | Orn_02 | 0.05 | 0.00 | **0.13** | 0.00 | 0.00 | 0.00 |
|  | Orn_11 | 0.03 | 0.00 | 0.00 | 0.00 | 0.01 | 0.00 |
|  | Orn_12 | 0.00 | 0.00 | 0.00 | 0.00 | 0.00 | 0.00 |
|  | Orn_16 | 0.00 | 0.00 | 0.00 | **0.17** | 0.00 | 0.00 |
|  | Orn_17 | 0.05 | **0.23** | 0.07 | 0.00 | **0.15** | 0.00 |
|  | Orn_18 | 0.00 | 0.00 | 0.00 | 0.00 | 0.00 | 0.00 |
|  | Orn_20 | 0.03 | 0.00 | 0.05 | 0.00 | 0.01 | 0.00 |
|  | Orn_21 | 0.03 | 0.00 | 0.06 | **0.11** | 0.05 | 0.03 |
|  | Orn_25 | 0.00 | 0.00 | 0.00 | 0.00 | 0.00 | 0.00 |
| **After correction** | Orn_01 | 0.00 | 0.00 | 0.00 | 0.00 | 0.01 | 0.01 |
|  | Orn_02 | 0.05 | 0.00 | 0.03 | 0.00 | 0.00 | 0.00 |
|  | Orn_11 | 0.03 | 0.00 | 0.00 | 0.00 | 0.01 | 0.00 |
|  | Orn_12 | 0.00 | 0.00 | 0.00 | 0.00 | 0.00 | 0.00 |
|  | Orn_16 | 0.00 | 0.00 | 0.00 | **0.17** | 0.00 | 0.00 |
|  | Orn_17 | 0.05 | **0.18** | 0.07 | 0.00 | 0.07 | 0.00 |
|  | Orn_18 | 0.00 | 0.00 | 0.00 | 0.00 | 0.00 | 0.00 |
|  | Orn_20 | 0.03 | 0.00 | 0.05 | 0.00 | 0.01 | 0.00 |
|  | Orn_21 | 0.03 | 0.00 | 0.06 | **0.11** | 0.05 | 0.03 |
|  | Orn_25 | 0.00 | 0.00 | 0.00 | 0.00 | 0.00 | 0.00 |
