# Supplementary material for: Oceanographic Currents and Local Ecological Knowledge Indicate, and Genetics Does Not Refute, a Contemporary Pattern of Larval Dispersal for The Ornate Spiny Lobster, Panulirus ornatus in the South-East Asian Archipelago
Source: PLoS One. 2015 May 7;10(5):e0124568. doi: 10.1371/journal.pone.0124568 (PMC4423998; doi:10.1371/journal.pone.0124568)
Supplement: S3 Table — http://dx.doi.org/10.5061/dryad.sp418/4. (DOCX) [file pone.0124568.s003.docx]

Table S3. Summary table of analysis of molecular variance (AMOVA) describing the partitioning of genetic variation for six *Panulirus ornatus* populations based on 10 microsatellite loci.

|  | **Source of variation (%)** | |  |  |
| --- | --- | --- | --- | --- |
|  | **Among population** | **Within population** | ***F_ST_*** | ***p- value*** |
| **In original dataset** | 0.26 | 99.74 | 0.003 | 0.195±0.004 |
| **After correction** | -0.04 | 100.04 | -0.000 | 0.571±0.005 |
