# Supplementary material for: Oceanographic Currents and Local Ecological Knowledge Indicate, and Genetics Does Not Refute, a Contemporary Pattern of Larval Dispersal for The Ornate Spiny Lobster, Panulirus ornatus in the South-East Asian Archipelago
Source: PLoS One. 2015 May 7;10(5):e0124568. doi: 10.1371/journal.pone.0124568 (PMC4423998; doi:10.1371/journal.pone.0124568)
Supplement: S4 Table — No significant value was found after correction using FDR. http://dx.doi.org/10.5061/dryad.sp418/5. (DOCX) [file pone.0124568.s004.docx]

Table S4. Genetic differentiation between *Panulirus ornatus* from collection locations using pairwise *F_ST_* for microsatellite loci in original dataset (lower value) and after correction (upper value). No significant value was found after correction using FDR.

|  | **Australia** |  | **Indonesia** | | |  | **Vietnam** | |
| --- | --- | --- | --- | --- | --- | --- | --- | --- |
| **Localities** | Torres Strait |  | West Timor | Lombok | North Sumatra |  | Binh Thuan | Da Nang |
| Torres Strait |  |  | 0.007 | -0.003 | -0.026 |  | 0.002 | -0.004 |
| West Timor | 0.006 |  |  | 0.008 | -0.014 |  | 0.005 | 0.012 |
| Lombok | -0.003 |  | 0.008 |  | -0.025 |  | 0.004 | -0.003 |
| North Sumatra | 0.029 |  | 0.030 | 0.034 |  |  | -0.021 | -0027 |
| Binh Thuan | 0.001 |  | 0.002 | 0.004 | 0.026 |  |  | 0.001 |
| Da Nang | 0.000 |  | 0.009 | 0.001 | 0.017 |  | 0.002 |  |
